# Neuromyelitis optica spectrum disorder with AQP4‐IgG presenting as area postrema syndrome and progressing to myelitis: A rare case report

**DOI:** 10.1002/ccr3.7636

**Published:** 2023-07-04

**Authors:** Preethi Jagannath, Mohammed Suhail K, Shaikh Mohammed Aslam S, Ashwin Kulkarni, Polasu Sri Satya Sai Prashanth, Hritik Madan, Ayush Anand

**Affiliations:** ^1^ M S Ramaiah Medical College Bengaluru India; ^2^ Department of General Medicine M S Ramaiah Medical College Bengaluru India; ^3^ Adesh Medical College and Hospital Kurukshetra Haryana India; ^4^ BP Koirala Institute of Health Sciences Dharan Nepal

**Keywords:** case report, diagnosis, management, neuromyelitis optica

## Abstract

**Key Clinical Message:**

Neuromyelitis optica spectrum disorders can less commonly present with area postrema syndrome progressing to myelitis. Management involves intravenous glucocorticoids, plasma exchange, and preventive immunotherapy.

**Abstract:**

Neuromyelitis optica spectrum disorders can less commonly present with area postrema syndrome progressing to myelitis. The majority of patients have positive AQP4‐Ab. Diagnosis is based on clinical and imaging findings. These patients can be treated with intravenous glucocorticoids, plasma exchange, and preventive immunotherapy.

## Graphical abstract

Neuromyelitis optica spectrum disorder with transverse myelitis.

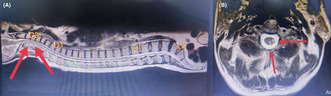

## 1 INTRODUCTION

Neuromyelitis optica spectrum disorders (NMOSDs) are rare immunological inflammatory disorders of the central nervous system (CNS) primarily involving the optic nerve, spinal cord, and brain.^1^, ^2^, ^3^ It is more common, has younger age of onset, has higher brain/brainstem involvement in Africans and Asians than Caucasians, and is usually diagnosed in middle‐aged females.^2^, ^4^ The majority of NMOSD patients have positive AQP4‐Ab.^1^ Diagnosis of NMOSD is based on International consensus diagnostic criteria, requiring certain clinical, laboratory, and imaging criteria to be met with the exclusion of differentials.^5^, ^6^ Acute attacks are primarily managed with intravenous corticosteroids.^1^ In worsening cases, plasma exchange may be indicated.^1^ Also, monoclonal antibodies, such as rituximab, eculizumab, tocilizumab, satralizumab, etc., can be used to modify the course of the disease.^7^, ^8^ Early diagnosis and intervention are crucial to prevent worsening and ensure optimum quality of life in these patients.^9^, ^10^ Herein, we present the case of a middle‐aged Asian male with NMOSD with AQP4‐IgG.

## 2 CASE REPORT

A 52‐year‐old Asian male presented with multiple episodes of hiccups, vomiting, decreased bladder sensation, and urinary retention for 3 weeks. In the past 2 weeks, he developed weakness in both lower limbs and reduced sensation in both lower limbs, followed by muscle spasms in both hands and legs. His past, personal, medical, and psychosocial history were unremarkable.

On presentation, he was conscious and oriented. On examination, blood pressure of 140/80 mm of Hg, pulse rate of 82 beats per minute, respiratory rate of 12 cycles per minute, and oxygen saturation of 98% on room air. Neurological examination (Table 1) revealed weakness in both lower limbs accompanied by the presence of the Babinski sign.

**TABLE 1:** Neurological examination of the patient.

Examination	Finding
Higher mental function	Normal
Cranial nerve examination	Normal
Motor examination	Muscle bulk and tone normal Power—upper and lower limb: 5/5 Hip flexion: bilateral grade 3/5 Hip extension: bilateral grade 3/5 Hip abduction: bilateral grade 4−/5 Hip adduction: bilateral grade 4/5 Knee flexion: bilateral grade 3/5 Knee extension: bilateral grade 3/5 Ankle dorsiflexion: grade 4+/5 Plantar flexion: bilateral grade 4−/5
Reflexes	Normal
Sensory	Impaired below the anterior superior iliac spine on both lower limbs
Joint position sense	Absent both lower limbs
Vibration	14 s both upper limbs Absent both lower limbs

**FIGURE 1:**
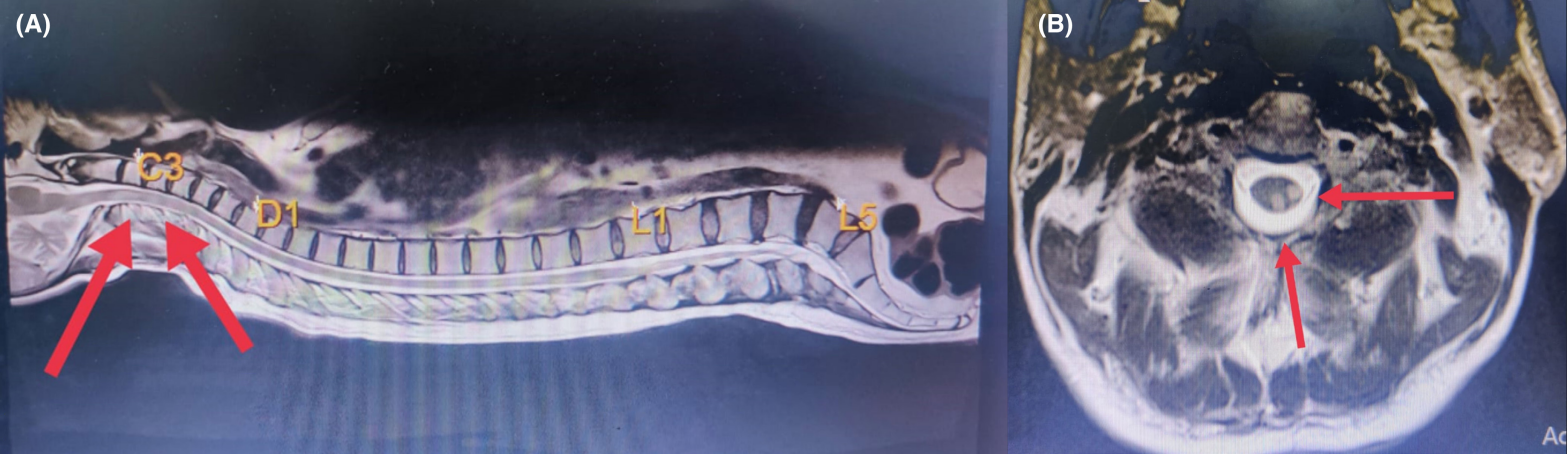
Transverse (A) and sagittal views (B) on spinal magnetic resonance imaging suggest acute transverse myelitis involving C3‐4.

Blood test results were normal, and cerebrospinal fluid examination revealed a high protein count with 5 lymphocytes, and the oligoclonal band was negative (Table 2). Serology for the anti‐herpes simplex virus, polymerase chain reaction analysis for herpes simplex virus, and cytomegalovirus were negative. The serum antibody AQP4 results were positive, and the Anti‐Mi2 antibody was borderline. Autoimmune antinuclear antibodies and anti‐DSA analysis were normal. Electrophysiological examination of visual evoked potential was normal. Ultrasonography (USG) of the abdomen showed a distended bladder with 250 mL post‐void residual urine. The spinal magnetic resonance imaging (MRI) revealed T2 hyperintensities with length measuring up to 18 mm and thickness of 8 mm noted predominantly in the left side of C3, C4, and another similar lesion measuring 12 × 6 mm in the C4 region in the posterior column of the cervical cord (Figure 1). Spinal MRI suggested acute transverse myelitis involving C3‐4. Brain magnetic resonance spectroscopy showed a description of a mild demyelination process. Brain MRI was unremarkable.

**TABLE 2:** Laboratory investigations of the patient.

Investigation	Result	Reference range
Hemoglobin (g/dL)	13.6	12–15
Platelets (cells/mm^3^)	112,000	150,000–400,000
Red blood cell count (million/mm^3^)	5.04	4.7–6.1
Serum creatinine (mg/dL)	1.3	0.62–1.10
Total leucocyte count (cells/mm^3^)	8800	4000–11,000
CSF investigations		
CSF cell count (cells/mm^3^)	5 Lymphocytes	<5 WBCs
Protein count (mg/dL)	72	15–60
Glucose (mg/dL)	56	50–75
Chloride (mmol/L)	123	115–130
Oligoclonal band	Negative	Negative

Abbreviation: CSF, cerebrospinal fluid.

NMOSD, multiple sclerosis (MS), and myelin oligodendrocyte glycoprotein antibody‐associated disease (MOGAD) were our patient's main differentials. We also considered systemic lupus erythematosus and infectious causes such as herpes simplex virus and cytomegalovirus. Based on the results of clinical symptoms and other investigations, the patient was diagnosed with NMOSD. The patient was started on intravenous methylprednisolone 1 g for 3 days and showed a 30% improvement in weakness in both lower limbs. Following this, he received 7 cycles of therapeutic plasma exchange (TPE) and physiotherapy. The patient was discharged after 14 days of hospital stay. Following discharge, mycophenolate mofetil was added with a monthly intravenous methylprednisolone pulse. The patient was symptomatically treated with baclofen, carbamazepine, and diazepam for recurrent painful muscle spasms. He experienced significant improvement in his neurological symptoms. The patient was on regular follow‐up until 6 months after discharge. During the regular follow‐up, the patient developed hypertension, which was managed with antihypertensives. On his last visit, he was counseled to repeat the spinal MRI. However, the patient could not afford it due to money constraints and was lost to follow‐up.

## 3 DISCUSSION

A meta‐analysis reported the prevalence of NMOSD ranges from 1.21 to 1.81 per thousand population.^3^ The annual incidence ranges from 0.135 to 0.420 per thousand population.^3^ Studies have found a slight female preponderance in NMOSD, with a maximum incidence and prevalence reported in 40–59 years of age.^2^, ^3^ Racial differences also exist in this disease. Studies have revealed that NMOSD is more prevalent in Africans and Asians than in the Caucasian population.^1^, ^3^ Also, race was significantly associated with brain/brainstem involvement and severity of attacks.^4^ Approximately 42% of Asians had brainstem involvement compared to 38% in the Afro‐American/Afro‐European population.^4^ Also, approximately 46% of Asians reported higher severity of the attack on disease onset than 38% of Caucasians.^4^ Similar to this, our patient was a middle‐aged Asian.

The diagnosis of NMOSD is made based on clinical, laboratory, and radiological findings. For diagnosing NMOSD with AQP4‐IgG, patients should have at least one of the six core clinical characteristics with AQP4‐IgG‐positive status and exclusion of alternative diagnosis.^5^, ^11^ Our patient presented with intractable hiccups and vomiting, progressing to motor involvement. This is a characteristic feature of area postrema syndrome (APS), which is the presenting feature in only one tenth of patients with NMOSD.^1^, ^12^ Furthermore, APS can progress to optic nerve involvement or myelitis. This makes this case presentation even more interesting, as our patient had a similar progression from APS to motor involvement. An MRI of the brain and spinal cord should be done to confirm CNS involvement.^5^ We also evaluated our patient with brain and spinal cord MRI. Brain MRI was unremarkable, as in most cases of disease onset, but spinal cord MRI revealed T2‐weighted hyperintensities in the C3‐4 region, suggesting acute transverse myelitis.^5^ Besides clinical and radiological findings, the serological investigation revealed AQP4‐IgG‐positive status. Despite this, ruling out MS and MOGAD is crucial in these cases. MS was ruled out based on MRI findings, a negative oligoclonal band in the CSF examination, and AQP4‐IgG‐positive status. Our setup did not have a MOG‐IgG testing facility, so we ruled out MOGAD based on MRI findings.

Acute attacks in NMOSD can be managed with intravenous glucocorticoids.^1^ In cases with worsening or no response to glucocorticoids, therapeutic plasma exchange can be used.^1^ Also, management of weakness, gait impairment, and bladder and bowel symptoms can significantly improve the quality of life in these patients.^1^, ^9^ We managed the acute attack with intravenous methylprednisolone followed by therapeutic plasma exchange. Like Aryal et al.,^13^ we managed painful spams with baclofen and diazepam. However, we used carbamazepine instead of eslicarbazepine based on availability. These interventions led to the successful management of acute attacks. Following this, we advised the patient for pulse therapy for methylprednisolone and mycophenolate mofetil. Though monoclonal antibodies are best for preventive immunotherapy, they are costly and are not readily procurable.^7^ Because of this, the patient was unable to afford it. Hence, we decide on mycophenolate mofetil, an alternative to monoclonal antibodies.^7^ These patients should be maintained on regular follow‐ups to detect relapses. However, the patient was lost to follow‐up after 6 months of diagnosis.

## 4 CONCLUSION

Diagnosis of NMOSD is based on International consensus diagnostic criteria within the exclusion of common differentials such as multiple sclerosis and myelin oligodendrocyte glycoprotein antibody‐associated disease. Less commonly, NMOSD with AQP4‐IgG can present with APS and progressive to myelitis. These patients should be managed with intravenous prednisolone, therapeutic plasma exchange, and preventive immunotherapy. In addition, long‐term follow‐up is required to detect relapses.

## AUTHOR CONTRIBUTIONS

**Preethi Jagannath:** Conceptualization; data curation; project administration; writing – original draft; writing – review and editing. **Mohammed Suhail K:** Conceptualization; data curation; supervision; writing – original draft; writing – review and editing. **Shaikh Mohammed Aslam S:** Conceptualization; data curation; project administration; supervision; validation; visualization; writing – original draft; writing – review and editing. **Ashwin Kulkarni:** Conceptualization; data curation; project administration; supervision; validation; visualization; writing – original draft; writing – review and editing. **Polasu Sri Satya Sai Prashanth:** Conceptualization; data curation; project administration; writing – original draft; writing – review and editing. **Hritik Madan:** Conceptualization; data curation; project administration; writing – original draft; writing – review and editing. **Ayush Anand:** Conceptualization; project administration; supervision; validation; visualization; writing – original draft; writing – review and editing.

## FUNDING INFORMATION

None.

## CONFLICT OF INTEREST STATEMENT

The authors have no conflict of interest to declare.

## CONSENT

Written informed consent was obtained from the patient to publish this report in accordance with the journal's patient consent policy.

## GUARANTOR

Dr. Shaikh Mohammed Aslam S is the Guarantor.

## Data Availability

All relevant data pertaining to this case are made available within the manuscript.
